# Spontaneous Pneumothorax: A 5-Year Experience

**DOI:** 10.4021/jocmr560w

**Published:** 2011-05-19

**Authors:** Cristiana Sousa, Joao Neves, Nuno Sa, Fabienne Goncalves, Julio Oliveira, Ernestina Reis

**Affiliations:** a Medicine Service, Unit D, Santo Antonio Hospital, Oporto Hospital Center, Porto, Portugal; b Medicine Service, Unit A, Santo Antonio Hospital, Oporto Hospital Center, Porto, Portugal

## Abstract

**Background:**

Spontaneous pneumothorax (SP) is defined by the presence of air in the pleural space without history of trauma. It is classified as secondary if coexisting with underlying pulmonary disease. Its an entity with considerable incidence and treatment particularities which give reason for a reflection on the subject. We present a 5-year casuistry, characterizing the SP epidemiology, clinical presentation, investigation and therapeutic choices.

**Methods:**

Sixty-six patients were included in the study, corresponding to 93 episodes of SP.

**Results:**

We have found male predominance and the mean age was 34.5 years old. In 60.6% of cases there was history of tobacco use; 36.4% of cases were classified as secondary; 30.1% of patients with secondary SP and 21.7% with primary SP recurred; 89.2% had an acute presentation. The most frequent initial symptom was chest pain (90.3%) and 81.7% had diminished breath sounds. In 17.3% it was documented a physical strain associated. We did not identify statistically significant association between the SP occurrence and the variation of the atmospheric pressure, on the first day of symptoms. In 12.9% of episodes the initial treatment option was observation. In most of the episodes the lung totally expanded. However, in 29.1% of the episodes surgical treatment was needed.

**Conclusions:**

Our results are similar to the literature. Some clinical records are incomplete, demanding the implementation of rules to improve knowledge about this matter.

**Keywords:**

Spontaneous pneumothorax; Primary spontaneous pneumothorax; Secondary spontaneous pneumothorax; Epidemiology

## Introduction

Spontaneous Pneumothorax (SP) is defined as the presence of air in the pleural cavity with the consequent collapse of the respective lung without an identified precipitating cause, namely a direct or indirect trauma, or iatrogenesis. The SP is said primary (PSP) in the absence of a chronic or acute pulmonary disease. The secondary (SSP) occurs more frequently related to chronic obstructive pulmonary disease (COPD), tuberculosis, cystic fibrosis or pneumocystosis [1-3].

The presence of air in the pleural cavity was primarily described in the 17th century by Riolan [4] in patients who suffered trauma. Hewson, in 1767 and for the first time, associated certain pulmonary pathologies to the presence of air in the pleural cavity, without evidence of trauma [4]. Itard, a student of Laennec, in 1803, was the first to designate this pathology/condition as Pneumothorax [5], but It was only in 1819 that Laennec described systematically its clinical aspects, clearly identifying the symptoms and findings in the objective examination [6].

If, on one hand, the knowledge acquisition on the physiopathology of the pneumothorax took place in the battlefields, on the other, the tuberculosis pandemic was the fuse to medical developments in the field of the spontaneous pneumothorax. As a matter of fact, the first definition of primary spontaneous pneumothorax implied the exclusion of trauma and Pulmonary Tuberculosis [4]. However, it was only in 1932 that Kjaergard elaborated the current definition of the primary SP as that occurring in healthy individuals [7].

Before the advent of the conventional pulmonary chest X-ray, the clinical diagnosis of SP in Pulmonary Tuberculosis patients was 10 times lower than that carried out at the time of the autopsy. With the massive use of the conventional pulmonary X-ray the identification of the SP became more frequent, this being thereon the complementary exam of choice for diagnosis [4].

The SP remains a frequent pathology nowadays, its incidence ranging between 18 - 28/100 000 per year in males and 1.2 - 6/100 000 per year in females [8, 9]. Currently, the health services provide care to an aged population with higher prevalence of lung diseases but also a longer life expectancy due to the therapeutic progresses of modern medicine. There are few review studies that characterize SP, from its clinical manifestations to treatment, in the current population, mainly in what concerns the Portuguese population.

The aim of the present work is to characterize the SP patients admitted in a medicine ward of a University Central Hospital. As such, we aim to contribute to a better knowledge of this pathology in our country, thus paving the way for new studies to be developed in this area.

## Methods

### Population and patients’ follow-up period

This is a retrospective study which took place in the Internal Medicine Service of the Santo Antonio Hospital, a university hospital.

All the patients admittted between the 1st January 2002 and 31st December 2006, whose discharge diagnosis was spontaneous pneumothorax, were included in the study.

### Characterization of sample

A review of the selected clinical files was carried out, demographic data was collected and the characterization of the population was done regarding comorbidities, risk factors, clinical manifestations, type of pneumothorax and number of relapses, therapeutic interventions and their complications. The atmospheric pressure data was provided by the Portuguese Institute of Meteorology.

### Statistical methodology

In order to carry out this retrospective study, a database was created in Microsoft Office Excel 2003 (version 11.0, part of Microsoft Office Professional Edition 2003 for Windows) and was treated for descriptive and inferential statistics. For the comparison of averages, Student’s t-tests were used (parametric test). The value 0.05 was assumed as the level of significance for the statistic tests carried out.

## Results

Seventy-one patients were identified but only 66 were included in the study. The remaining 5 were excluded due to lack of access to the clinical data. Ninety-three episodes of SP were characterized.

**Table 1:** Age and Gender Distribution per Type of SP

Type of SP	Total	Gender		Mean age (pattern deviation)
	%	Male %	Female %	Years
Primary SP	63.6	76.2	23.8	30.1 (± 15.2)
Secondary SP	36.4	83.3	16.7	48.5 (± 21.6)

The male gender was the prevailing (male : female ratio, 3.7 : 1). Age varied between 13 to 82 years old (mean 34.5 ± 18.8 years old), the females being on average older 2.8 years. Table 1 presents the age and gender distribution per type of SP.

Sixty point six percent (60.6%) of the subjects had current or past history of tobacco use. Seven used concomitantly other inhaled or smoked drugs (mostly hashish), and one non-smoker only used cannabinoids. Only two females did not smoke.

**Table 2:** Type of Pulmonary Disease

Pulmonary disease	N
Lung emphysema not related to smoking	3
Idiopathic pulmonary fibrosis	3
COPD	4
Asthma	2
Lung cancer primary/secondary neoplasia	5
Sarcoidosis	1
History of Pulmonary Tuberculosis/Pulmonary Tuberculosis sequels	9
Bronchiectasis	2
Cystic fibrosis	2

In 36.4% of the mentioned cases there was an underlying pulmonary disease, having therefore been classified as secondary (Table 2). Eighty-one percent were male.

For the patients with PSP subject to VATS, thoracoscopy or thoracotomy, subpleural bullae were observed in 73.3% of the cases.

No correlation was found with the atmospheric pressure or with its variance on the day the complaints onset (Student’s t-test, P > 0.05).

In 30.1% of the secondary SP patients and 21.7% of the primary SP patients there was a relapse. The maximum number of relapses per patient was 4 and the average time between each recurrence was 21.9 months. The relapse was more frequent in females (42.9% vs. 19.2%).

**Table 3:** Activity When Symptoms Onset

Type of activity	N	%
Rest	29	31.2
Coughing	5	5.3
Effort (type not specified)	2	2.2
Work activity (type not specified)	3	3.2
Upon waking up	5	5.3
Respiratory Cinesitherapy	1	1.1
Not mentioned	48	51.6
Total	93	100

The presentation was sudden in 89.2% of the cases. The ongoing activity when the symptoms started is shown in Table 3. It should be noted that 31.2% of the cases occurred at rest and that 51.6% of the cases had no reference in clinical file, as to the activity at the time the symptoms started.

**Table 4:** Symptoms and Signs

Symptoms		N	%
Pain	Ipsilateral	74	79.6
	Retrosternal	6	6.4
	Posterior	1	1.1
	Location not described	3	3.2
Dyspnea	Grade I	8	8.6
	Grade II	8	8.6
	Grade III	4	4.3
	Grade IV	14	15
	Non-characterized	20	21.5
Dry cough		40	43
Asymptomatic		1	1.1
**Signs**			
Tachypnea		35	37.6
Tachycardia		26	28
Hypertension		16	17.2
Hypotension		2	2.2
Desaturation		11	11.8
Decreased breath sounds		76	81.7
Hyper-resonance		11	11.8
Subcutaneous emphysema		6	6.6

In Table 4, we describe the most frequent symptoms and signs.

Except for one patient, the SP was diagnosed through conventional X-ray. All the SPs were unilateral and most of them occurred on the left side (59.1%).

For 89.2% of the cases, the SPs were free.

**Table 5:** Result of Blood Gas

	N	%
Normal	32	34.4
Hypoxemia	12	12.9
Respiratory failure type 1	10	10.8
Respiratory failure type 2	4	4.3
Not conducted/not recorded	35	37.6

No hypertensive SP was identified and 4.3% of cases had pleural effusion associated. In 62.4% there was record of blood gas analysis, the results observable in Table 5.

**Table 6:** Type of Treatment

**Type of treatment**			N	%
Analgesia			84	90.3
Oxygen therapy			86	92.5
Observation			12	12.9
Simple aspiration			7	7.5
Chest tube			80	86
Chemical pleurodesis			1	1.1
Type of surgery (elective and non-elective)				
Thoracotomy	Bullectomy	3	8	8.6
	Lobectomy	3		
	Atypical	2		
Simple Thoracoscopy		1		1.1
Video Assisted Thoracoscopy	Bullectomy	5	19	20.4
	Lobectomy	2		
	Atypical	10		
	Unknown	2		

In Table 6, we present the types of treatment conducted. Only 65.6% of the cases had express order to rest in the clinical file. The initial treatment did not include invasive acts for 12.9% of the cases. When these were necessary, simple aspiration was used for 7.5% and a chest tube for 86% (78.5% on the admittance day). Low vacuum pressure was placed in 47.3% and one patient underwent chemical pleurodesis by chest tube. 

**Table 7:** Simple Aspiration or Chest Tube Complications

Complication	N	%
Pain at the insertion site	25	28.7
Infection on the insertion site	2	2.3
Hemorrhage/hemothorax	3	3.4
Hypotension	2	2.3
Subcutaneous emphysema	6	6.9
Malfunction of chest tube	15	17.2

Table 7 presents the most frequent chest tube complications.

The average hospitalization period was 8 days (from 1 to 32 days).

There was complete re-expansion of the lung in 71% of the cases and 23.7% were referred directly to surgery during the hospitalization, due to persistent gas fistula. Three deaths were recorded, but without direct correlation to the SP.

## Discussion

To the initial fervor, when descriptive studies multiplied focusing mainly on the pathophysiology and the epidemiology of the pneumothorax, a period of mild interest by the topic followed. The development of new therapeutic strategies re-launched the interest in the matter.

The environmental changes and also those in the habits of the population, as well as improvements in health care led to believe that the results of these studies no longer applied to the current reality. However, recent reviews [3, 10] do not reveal significant differences.

Our study has showed us a population with characteristics similar to those in international studies. We encountered a population with PSP that was mostly young and male, the occurrence peak being later when there was an underlying structural pulmonary disease (SSP) [3, 4]. Although usually found in tall and thin individuals [11], the data concerning the patients physical built was not described in our research.

According to Ferraro and colleagues, the PSP corresponds to 80% of the SP and only 20% have an underlying pulmonary disease [12] and Weissberg found a similar relation (70% vs. 30%) [1]. Nevertheless, other authors such as Light describe an almost equal relation; these differences should explain population from different studies [13]. In our sample, the distribution of PSP/SSP is within these ranges.

The pathogenesis of the pneumothorax is controversial, mainly in the PSP. The presence of bullae is described in up to 90% of the patients who underwent thoracoscopy or thoracotomy, and 80% in the chest CT scan [14, 15]; the rupture of these bullae into the pleural space seems to be intimately involved with the genesis of the SP. However, the data on the presence of these changes in the general population are scarce. In our study, bullae/blebs were described in 73.3% of the patients subject to VATS, thoracoscopy or thoracotomy, a value not discrepant from the one described in the literature.

On the other hand, it is believed that the rupture of these bullae is related to the changes in the transpulmonary pressure gradient and its variation in particular situations (such as the retention of secretions/bronchiolitis [2], diving [16], air travel [17]). The impact of the atmospheric pressure variations in the occurrence of SP was also researched in several studies [18-22] and it is controversial. Bense [21] found a statistically significant relation between an atmospheric pressure drop of 10 milibars and the number of admissions on the two subsequent days and Scott [22] described that 72% of the patients hospitalized due to PSP had been exposed to an unusual variation of atmospheric pressure on the four previous days. Nevertheless, studies carried out by Suarez-Varel et al [23] and Smit et al [20] did not find a relation with statistic significance. These discrepancies may be the result of differences in the study design, population and location of the meteorological stations regarding either the hospital or the patient’s residence [19]. In our study, we have not found a statistic significant relation either, possibly by the multifactorial nature of the process.

It is also frequently assumed that the physical effort can promote SP. However this relation was never really substantiated [15, 24, 25]. In our research, it was not possible to determine it, because the physical activity was not described in a considerable number of episodes at the onset of the symptoms. For those who were referred, most (64.4%) were at rest. In studies such as Weissbergs only 10% of the episodes took place with relation to physical effort [10].

The association with smoking is recognized [26]. The guidelines of the British Thoracic Society recommend emphasis on the relation between a relapse of the SP and tobacco use, in an attempt to encourage the patients to quit smoking [27]. In our series, most of the patients had a history of present or past use of tobacco, but a statistically significant relation with the risk of relapse was not found. In our population, only a small percentage of the subjects were referred to smoking cessation consultations.

The recurrence rate for the PSP ranges from 16 to 52% and for the SSP from 39 to 47%, most of them taking place between six months to two years after the first episode [28]. The present study found a PSP recurrence rate lower than described, fact which may have been distorted by the small dimension of the sample and loss of follow up data. The mean time in our study was longer, because it included a patient who had a relapse twenty years after the first episode (except for this patient, the mean time would be 12.3 months).

The beginning of the SP is generally characterized by ipsilateral pleuritic pain or sudden dyspnea and the most recurrent findings, at the objective examination, are the decrease of breath sounds, hyper-resonance and tachycardia [3, 10]. These were also the most common ones in our study.

While the history and clinical examination may suggest the presence of pneumothorax, the diagnosis is generally made through conventional X-ray [28]. This was also the standard diagnosis method for our study.

The handling of the SP is meant, on one hand, to remove the air from the pleural space and on the other, to avoid relapses [3]. The therapeutic options range from simple observation, simple aspiration, placement of the chest tube, pleurodesis, thoracoscopy, video assisted thoracoscopic surgery and thoracotomy. The choice regarding the method to be used depends on factors such as the intensity of the symptoms, the pneumothorax size, duration and persistence of the gas fistula and its classification as primary or secondary [3].

The simple observation is only recommended for the small and mildly symptomatic PSPs and it is rarely recommended for the SSP, being applied only when these are small and have with mildly symptomatic [28]. As would be expected in our study, most SPs, where the initial option was conservative, were PSPs (75%). VATS and thoracoscopy are more frequently applied to the SSP cases, whose treatment is widely known to be more difficult.

The oxygen supplementation accelerates the re-absorption of the pneumothorax [29], and is therefore recommended to all the hospitalized patients [28]. For 7.5% of our patients, oxygen was not prescribed.

Simple aspiration is recommended as a first line treatment for all the PSPs which have indication for intervention and for the SSPs only when these are of small volume, have few symptoms and occur in individuals that are less than 50 years old. The chest tube is recommended whenever the simple aspiration fails and also for most of the SSPs [28]. Our research shows that there is an overuse of the chest tube rather than the simple aspiration, mostly in the PSPs. This implies a bigger risk of complications and length of hospitalization. In our series, 45% of the patients had complications related to the chest tube, a rate two-fold higher than that found by Chan and colleagues, who described a rate of 18% and out of these only 4% were connected with the bad placement of the chest tube [30]. This rate is considerably influenced by the appreciation of clinical pain. Our study did not aim to examine in detail the reason/motive for the bad functioning of the chest tube but in some cases it was attributed to the excessive length of the intra-thoracic portion of the drain.

Our study was at the same time an auditing exercise, which allowed us to verify some flaws in our clinical records, mainly those conducted in the emergency service, where the description of the objective examination revealed itself poor. Other missing data in most of the clinical processes were: the activity at the time of the symptoms onset, the patients height and weight. Concerning the treatments provided, the moment of the therapeutic intervention was not always clear and for most of the patients undergoing surgery outside our institution there was no information regarding the procedure or its outcome.

The clinical records reflect, to some extent, our level of care which calls on a reflection about this data and the implementation of measures which may allow their improvement.
